# P304: Conducts following occupational accidents involving exposure to biological material among emergency medical services personnel

**DOI:** 10.1186/2047-2994-2-S1-P304

**Published:** 2013-06-20

**Authors:** MHRS Paiva, ACD Oliveira, AOD Paula

**Affiliations:** 1 Universidade Federal de Minas Gerais, Belo Horizonte / Minas Gerais, Brazil

## Introduction

The adoption of post-accident conducts can be decisive for minimizing or avoiding the acquisition of diseases as a result of the occupational accident.

## Objectives

The aim was to estimate the prevalence of accidents involving exposure to biological material, post-accident conducts and the undertaking of serological monitoring concerning the possibility of transmission of the AIDS virus and Hepatitis B and C among the Emergency Medical Services professionals in Minas Gerais, Brazil.

## Methods

This descriptive cross-sectional study was undertaken with professionals of the public EMS in the state of Minas Gerais. Data was collected between December 2011 and July 2012, via a structured questionnaire and analyzed by the Statistical Package for Social Science (SPSS). Characterization of population, post-accident conducts and serological monitoring of the affected professional was verified through calculation of absolute and relative frequencies.

## Results

487 workers participated in the study; 124 physicians (25.5%), 60 nurses (12.3%), 173 nurse technicians (35.5%) and 130 drivers (26.7%). The global prevalence of professionals occupationally involved in biological material accidents was 17.0 % (83/487). However, 33.7% of these workers referred to more than one accident, totaling 121 exposures to body fluids in this period. It was ascertained that after the accident, 35.5% (43/121) of the cases reported undergoing medical evaluation; for 29.7% (36/121) an accident report was issued; for 13.2% (16/121), 9.1% (11/121) and 10.7% (13/121) the undertaking of serology for Hepatitis B, C and HIV respectively was mentioned, both from departments and personnel. For Hepatitis B it was ascertained that only 4.9% (6/121) of affected professionals were monitored for one year after the occurrence of exposure to BM; 2.5% (03/121) for Hepatitis C; and 5.8% (07/121) for HIV.

## Conclusion

It is hoped that these results may stimulate discussion about the importance of accident notification and evaluation, and of the monitoring of the affected professional.

## Disclosure of interest

None declared

